# Presence of *Anaplasma phagocytophilum* Ecotype I in UK Ruminants and Associated Zoonotic Risk

**DOI:** 10.3390/pathogens12020216

**Published:** 2023-01-30

**Authors:** Laura Bianchessi, Mara Silvia Rocchi, Madeleine Maley, Kayleigh Allen, Keith Ballingall, Lauretta Turin

**Affiliations:** 1 Department of Veterinary Medicine and Animal Sciences, University of Milan, Via dell’Università 6, 26900 Lodi, Italy; 2 Moredun Research Institute, Pentlands Science Park, Bush Loan, Penicuik EH26 OPZ, UK

**Keywords:** *Anaplasma phagocytophilum*, tick-borne pathogen, ecotype, reservoir, phylogeny, emerging zoonosis, One Health, surveillance, ruminants, tick-borne fever

## Abstract

*Anaplasma phagocytophilum* is the causative agent of tick-borne fever in sheep, pasture fever in cattle, and granulocytic anaplasmosis in humans. The increasing prevalence and transboundary spread of *A. phagocytophilum* in livestock, ticks, and wildlife in the UK poses a potential zoonotic risk that has yet to be estimated. Several ecotypes of *A. phagocytophilum* show variable zoonotic potential. To evaluate the possible risk associated with the transmission of *A. phagocytophilum* from ruminants to humans, the ecotype was determined by sequencing the *groEL* gene from 71 positive blood and tissue samples from UK ruminants. Thirty-four *groEL* sequences were obtained, fourteen of which were identified in multiple samples. Of the 13 nucleotide polymorphisms identified through pairwise comparison, all corresponded to synonymous substitutions. The subsequent phylogenetic estimation of the relationship with other European/world isolates indicated that all the *groEL* sequences clustered with other ecotype I sequences. The presence of ecotype I closely reflects that observed in ruminants in continental Europe and suggests a lower risk of zoonotic transmission from this reservoir.

## 1. Introduction

Zoonoses from tick-borne pathogens have increased dramatically in the last few decades [1] and now pose a serious problem worldwide, due to their impact on public health and livestock production, as well as increased morbidity in wildlife [2]. Climate change, globalization, population movements and growth, changes of landscapes and natural habitats, and shifts in host geographic range and population density have led to the emergence of diverse ecotypes of the tick-transmitted bacterium *Anaplasma phagocytophilum* [3,4].

*Anaplasma phagocytophilum* is a Gram-negative bacterium (family Anaplasmataceae, order Rickettsiales) which infects lymphoid cells of the immune system (neutrophils and other cells of myeloid and non-myeloid origin) causing mild-to-severe immunosuppression in vertebrate hosts, including humans. *Anaplasma phagocytophilum* is tick-transmitted and can manifest with different presentations, from a mild febrile-like illness to a severe disease, which can be complicated by secondary infections, and in some species can progress to abortion. Outbreaks of disease associated with *A. phagocytophilum* infection in ruminants are increasingly being reported in the UK (http://apha.defra.gov.uk//vet-gateway/surveillance/scanning/disease-dashboards.htm accessed on 18 January 2023). *Anaplasma phagocytophilum* can infect several animal species, including sheep (*Ovis aries*), cattle (*Bos taurus*), goat (*Capra aegagrus hircus*), dog (*Canis lupus familiaris*), horse (*Equus caballus*), red deer (*Cervus elaphus*), roe deer (*Capreolus capreolus*) and white-tailed deer (*Odocoileus virginianus*) as well as wild boar (*Sus scrofa*), wild rodents, hedgehogs, birds, and humans [4]. The role of the tick vectors and different vertebrate hosts as reservoirs of infection remains unclear [5] in our understanding of disease epidemiology and zoonotic risk [6]. For example, ruminant infections are widespread across Europe but not yet reported in the USA, whereas human, equine, and canine infections have been reported worldwide [7].

Transmission of *A. phagocytophilum* involves many species of *Ixodes* ticks (*I. scapularis*, *I. pacificus*, *I. spinipalpis*, *I. ricinus*, *I. persulcatus*, *I. ovatus*), with only a small number of additional vectors (e.g. deer keds, *Lipoptena cervi*) possibly also involved [4]. In Europe, the main vector is *I. ricinus* [3,8]. Ticks acquire the bacterium from infected vertebrate hosts through a blood meal, and the infection is maintained through trans-stadial transmission, including a trans-ovarian stage [9]. *Anaplasma phagocytophilum* is known to survive in the salivary glands and midgut cells of the infected ticks, which can transmit the bacterium to other vertebrate hosts during the blood meal. 

*Anaplasma phagocytophilum* is characterized by a high degree of genetic diversity, resulting in variations in pathogenicity and host tropism [6]. Indeed, several studies have demonstrated that different strains are characterized by different host range predilections, and not all strains can infect all hosts [10,11,12]. Due to the existence of diverse genetic variants, vectors, and vertebrate hosts, the ecoepidemiology of the infection caused by *A. phagocytophilum* is complex, also involving distinct cycles in various geographical locations [13,14].

Several approaches, targeting different genes, have been used to differentiate *A. phagocytophilum* variants into different clades, namely, ecotypes, clusters, and haplotypes [14]. Initially, Jahfari et al. [14] proposed a division into four ecotypes, based on the sequence of the *groEL* heat shock operon gene, which are linked to different combinations of mammalian hosts, tick vectors, and geographical regions (Table 1). However, the *groEL* gene is relatively conserved compared to other markers and does not always show enough discrimination power to segregate some clusters, specifically when short fragments of the gene are analysed. Therefore, more recently, Jaarsma et al. [15] and Grassi et al. [16] further subdivided the four ecotypes identified by Jahfari et al. into clusters and haplotypes (Table 1). Nevertheless, the subdivision into ecotypes is still preferred as it is simpler and has been extensively used by other authors especially from European countries [17,18].

Based on the diversity present in the *groEL* gene, in Europe, multiple ecotypes of *A. phagocytophilum* are reported to circulate in mammals in three enzootic cycles, one primarily infecting nest-living mammals such as voles and shrews [12,14,15], one host-generalist spreading in livestock, companion animals, and humans [14,19], and a third host-specialist infecting primarily roe deer [20,21]. The host-generalist and host-specialist ecotypes, which are genetically similar, are transmitted by *I. ricinus*, while *A. phagocytophilum* infecting burrowing mammals is genetically distant and is mainly transmitted by *I. trianguliceps* [12,20,21]. Overall, ecotype I (zoonotic) is associated with cattle, horses, mouflons, small ruminants, hedgehogs, red deer, and humans, whereas ecotype II is linked only to ruminants and particularly roe deer; ecotype III is related to rodents, and ecotype IV is associated with birds [14,15]. Other proposed differentiations include clustering based on the sequence of the transcription regulatory protein *ankA* gene or the major surface protein 2 and 4 genes (*msp2* and *msp4*) [13,14,16]. 

Few studies have investigated *A. phagocytophilum* prevalence in the UK. A recently published study on questing ticks revealed a higher prevalence in the recreational area of Northern England (4.7%, corresponding to 38/831 positive nymphs) than in Southern England (1.8%, 44/2385), with the majority (87%, 99/114) of strains belonging to *groEL* ecotype I and the remaining (13%, 15/114) to *groEL* ecotype II; only ecotype I was detected in Wales [22]. The Welsh study also revealed a correlation between sheep grazing and a higher prevalence of *A. phagocytophilum*. Another recently published study, still on questing ticks, revealed a 4.7% prevalence of *A. phagocytophilum* in Wester Ross, the northwest area of the Scottish Highlands, with *A. phagocytophilum* being the most prevalent tick-borne pathogen detected. According to the authors, the majority of the strains (86%) were identified as the zoonotic ecotype I, probably maintained by red deer, and the remaining (14%) as the non-zoonotic ecotype II, probably maintained by roe deer [23]. An eco-epidemiological screening of the rodent community in West Wales revealed a low prevalence of *A. phagocytophilum,* which was detected only at one site in ticks collected from bank voles [24]. To our knowledge, there are no published studies elucidating the ecotypes of *A. phagocytophilum* present in UK ruminants.

*Anaplasma phagocytophilum* causes tick-borne fever (TBF) in sheep, pasture fever in cattle, and granulocytic anaplasmosis in humans (HGA), equines (EGA), and canines (CGA) [4,13]. In both sheep and cattle, infections with *A. phagocytophilum* have a considerable economic impact due to diminished fertility, increased abortions, and lowered milk production and, sometimes, can result in death [4]. Such infections also provide a reservoir for genetic diversification and the spread of the pathogen [8]. *Anaplasma phagocytophilum* also causes immunosuppression in its host and, consequently, increased incidence of secondary infections by opportunistic pathogens, with outbreaks of diseases such as tick pyemia due to *Staphylococcus aureus*, pneumonia due to *Pasteurella multlocida, Trueperella pyogenes* or opportunistic fungi, septicaemic listeriosis, fatal encephalitis due to *Louping ill* virus, severe orf (contagious ecthyma), and other unusual disease presentations [13,25,26]. Humans are often exposed to ticks and, consequently, to strains of *A. phagocytophilum* with zoonotic potential. The increasing prevalence of *A. phagocytophilum* in ticks, livestock, and wildlife is compounded by the lack of vaccines and limited treatment options [4]. Consequently, the characterization of *A. phagocytophilum* strains in clinical samples and ticks will improve our understanding of the host preference, geographical segregation of ecotypes, and the zoonotic potential of each strain. The aim of this study was to investigate the genetic diversity of the tick-borne pathogen *A. phagocytophilum* in samples derived from UK ruminants to allow the prediction of its pathogenicity and vertebrate host specificity, as well as to infer its zoonotic potential.

## 2. Materials and Methods

### 2.1. Sample Preparation and DNA Extraction

Seventy-one tissues and uncoagulated blood samples were submitted between 2020 and 2022 to the Virus Surveillance Unit at the Moredun Research Institute for investigation using an *A. phagocytophilum* real-time qPCR targeting a 77-bp fragment of the *msp2* gene [27]. Samples originated from the Animal and Plant Health Agency Veterinary Investigation Centres, the SRUC Disease Surveillance Centres, and from private veterinary practitioners. Ethical review and approval were waived for this study due to the nature of the samples and the anonymization of results. All the isolates and fresh samples for *A. phagocytophilum* isolation originated from clinical diagnostic or pathology submission, where the blood/tissues were collected for non-research purposes as an act of veterinary surgery. The Virus Surveillance Unit submission form specifically requests consent for anonymous surplus sample use, which was granted in all cases. The majority of the samples were represented by ovine spleens (n = 43), followed by ovine EDTA blood (n = 19). A summary of the characteristics of the samples tested is shown in Table 2. Tissue homogenates in Viral Transport Media and buffy coats from uncoagulated blood were processed according to standard protocols. Total DNA extraction was performed using the DNeasy^®^ blood and tissue kit (Qiagen, Hilden, Germany) according to manufacturer’s instructions. DNA quality was checked using a NanoDrop one microvolume spectrophotometer (ThermoFisher Scientific, Waltham, MA, USA). Positive control DNA samples were prepared from the *A. phagocytophilum* feral goat strain (APFG) provided by the Tick Cell Biobank, University of Liverpool [28]. Samples and positive control were tested using qPCR before use for quality control purposes.

### 2.2. PCR for the Amplification and Sequencing of the groEL Gene

Several primers previously described [29] were investigated to target a 573–666 bp fragment of the *A. phagocytophilum groEL* gene to select the most sensitive and specific approach for PCR amplification, Sanger sequencing, and phylogenetic analysis. In addition to *A. phagocytophilum,* some of the primers also anneal with other bacterial targets, such as *A. platys*, *A. bovis*, *Ehrlichia* sp., *Lentilitoribacter* sp., *Neorickettsia findlayensis*, *Rhodobiaceae bacterium*, *Erythrobacter* sp., and *Cohaesibacter* sp., and contain degenerate basis. To increase specificity, a second set of non-degenerate primers modified to anneal only to *A. phagocytophilum* sequences was also tested. The primers selected for this work are shown in Table 3. The following primer combinations were used to test the ruminant samples: 569:1236 and nd643:nd1236. PCR was carried out using the HotStarTaq DNA Polymerase^®^ kit (Qiagen, Hilden, Germany) according to manufacturer’s instructions and containing 3 μL of DNA template. PCR reactions were carried out in a final volume 50 μL to allow electrophoretic analysis, as well as successive fragment isolation. PCR cycling conditions were 10 m at 95 °C, followed by 45 cycles of 1 m at 95 °C, 30 s at 57 °C, and 45 s at 72 °C, with a final elongation of 10 m at 72 °C. PCR products were visualised on a 1.5% TAE agarose gel according to standard procedures. Amplicons were gel-purified using the ChargeSwitch™ PCR clean-up kit (Thermo Fisher) according to manufacturer’s instructions and Sanger sequenced bi-directionally using Eurofins MWG (https://eurofinsgenomics.eu/en/custom-dna-sequencing/additional-services/sample-submission/ accessed on 18 January 2023).

### 2.3. Nucleotide Sequence Analysis

Nucleotide sequences were analysed using SeqMan Pro 17 (DNAStar Lasergene software V17) and BioEdit (V 7.2) software. Following primer removal, a consensus sequence was generated for each of the two primer combinations (569:1236 and nd643:nd1236, respectively); these consensus sequences were aligned to create a final validated consensus sequence representing the product of the four sequencing reactions. The sequences obtained in this study have been deposited in GenBank under accession numbers OQ060727- OQ060798.

The final consensus sequences from each sample were aligned using MegAlign Pro 17 (DNAStar Lasergene software, V17) to identify nucleotide variations. Sequences showing nucleotide variations were translated into amino-acid sequences to identify synonymous and nonsynonymous substitutions using SeqBuilder Pro 17 (DNAStar Lasergene software, V17).

### 2.4. Phylogenetic Analysis

In total, 1082 *groEL* sequences, including examples of all four ecotypes, were extracted from the NCBI database and previously published literature [14,15]. A multiple alignment of these sequences was generated using Clustal Omega, (https://www.ebi.ac.uk/Tools/msa/clustalo/ accessed on 18 January 2023). Duplicate sequences were removed, and the remaining 412 *groEL* sequences, including the newly identified sequences from this study, were re-aligned, then used to generate a maximum likelihood tree in IQ-TREE [31]. The model selection tool in IQ-TREE [32] was employed to choose the optimum substitution model, which was GTR+F+R5 [33]. The ultrafast boot strap method of Minh et al. [34] was used to test tree topology. 

A second, simplified phylogenetic tree was prepared for illustration purposes, by removing all the non-European sequences. This resulted in an alignment of 153 sequences, of which 34 were the ones described in this paper. In this case, the substitution model selected was GTR+F+R3. Phylogenetic trees were prepared for publication by importing into Dendroscope 3.8.4 [35]. 

Finally, to attempt to relate sequence variation of our isolates to ecotype I clusters previously identified as potentially zoonotic, we selected all ecotype I sequences from the initial 1082 extracted and aligned these with those generated in this study. Sequences were aligned in Clustal Omega as described above, and the model selected in IQ-TREE was TN+F+R3. The tree was exported in Newick format and uploaded to Dendroscope for graphical editing. 

## 3. Results

### 3.1. Primer Selection and groEL PCR Assays on Ruminant Samples

The primer combinations to be used for further analysis were selected based on the quality and specificity of the amplification and quality of the subsequent nucleotide sequence. Both primer combinations tested (degenerate and non-degenerate) showed good amplification of the positive samples and were considered equivalent. All samples in this study were tested with both PCR assays (degenerate and non-degenerate) and produced high quality sequences, with only three samples failing one or the other PCR assay. Forty-four out of forty-six tissue samples and twenty-four out of twenty-five blood samples were positive in both PCRs. For the samples which failed one of the two PCR assays, sequence analysis was, therefore, carried out only on one set of PCR products. There was no difference in PCR performance or sequence quality between matrices or host animal species.

### 3.2. Sequence Analysis

All purified PCR products yielded high quality sequences that confirmed the identity of all the PCR products and revealed between 99 to 100% sequence identity to previously deposited *A. phagocytophilum groEL* reference sequences: AF548386.1, EU246959.1, HM057224.1, HM057231.1, HM057232.1, HM057233.1, KC800986.1, KF312357.1, KF312358.1, KF312359.1, KF312360.1, KF312361.1, KF383241.1, KM215262.1, KM215265.1, MT498616.1, MW732492.1, and OM127910.1. All sequences were trimmed to exclude primers and standardised to a 571 bp amplicon. Alignment of the consensus fragments obtained using the two PCR assays for the same sample produced the final consensus sequences (one per sample). Three out of seventy-two sequences showed a single base difference between the two PCR assays (Supplementary Figure S1). These differences were recorded using the IUPAC coding system for nucleotide nomenclature and retained for the phylogenetic analysis. 

We obtained sequence data for all 72 samples tested (71 samples and 1 positive control obtained from the tick-cell biobank). In total, 20 samples yielded sequences represented only once in this dataset, whereas in the remaining 52 samples, we identified 14 sequences which appeared in 2 or more samples. Three sequences (OQ060730, OQ060733, and OQ060739) were found in nine, six, and seven samples, respectively. No duplicate sequences were found in samples originating from the same flock or herd and submitted for testing at the same time. 

The alignment of the sequences obtained from all samples tested also identified 13 positions where nucleotide variations (polymorphisms) were present, with 4 found only once, while others were found in up to half of the sequences analysed. When compared to the reference coding sequence (Accn. N. JQ685509) all 13 polymorphic sites represented synonymous substitutions (Supplementary Table S1). 

### 3.3. Phylogenetic Analysis

**Figure 1:**
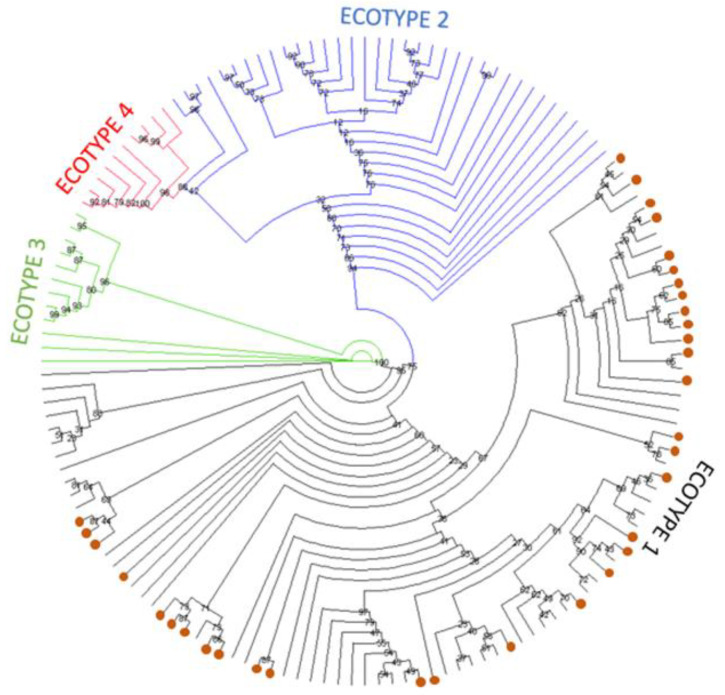
Simplified maximum likelihood phylogenetic tree presented as a circular cladogram showing the relationship between the 34 *groEL* sequences generated in this work and representative European sequences from ecotypes I-IV. Phylogenetic analysis was performed using the GTR+F+R5 model in IQ-TREE with 1000 bootstrap replicates. Sequences generated in this work are shown as orange dots, and colours are used to separate sequences in the different ecotypes. Bootstrap values are shown next to the nodes of the trees. The final data set contained 449 positions, and the corresponding tree is shown in Supplementary Figure S2.

Phylogenetic estimation of the relationship between the *groEL* sequences identified in this study and those representing each of the four previously identified ecotypes (Supplementary Table S2) is shown in Figure 1 as a simplified tree. The complete tree is shown in Supplementary Figure S2. The tree estimates that all 34 sequences identified in this study cluster with sequences previously defined as ecotype I in the European samples.

**Figure 2:**
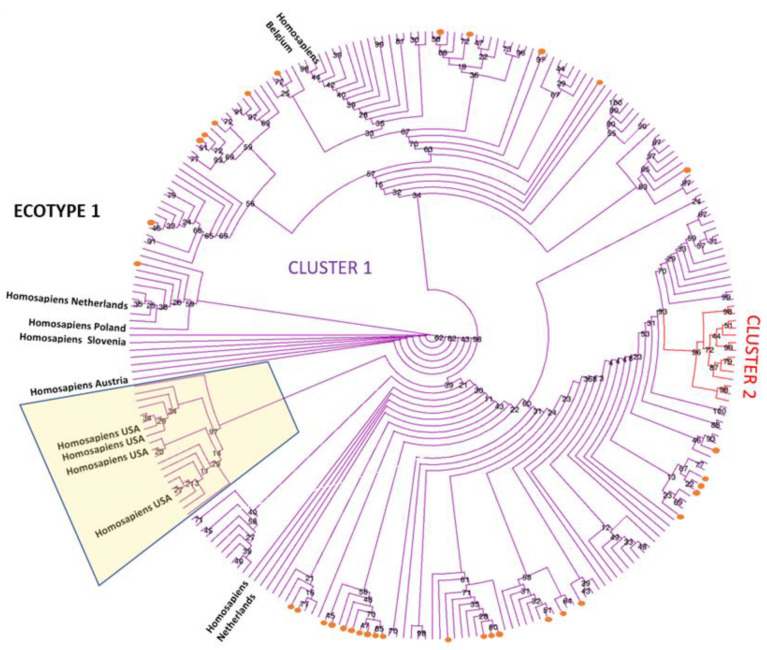
Maximum likelihood phylogenetic tree presented as a circular cladogram showing the relationship between the 34 *groEL* sequences generated in this work and representative ecotype I sequences. Phylogenetic analysis was performed using the TN+F+R3 model in IQ-TREE with 1000 bootstrap replicates. Sequences generated in this work are shown as orange dots, and colours are used to differentiate sequences in the different clusters. Bootstrap values are shown next to the nodes of the trees. The final data set contained 260 positions. Human sequences derived from samples from the United States are located in the shadowed box.

The phylogenetic estimation of the relationship between the *groEL* sequences identified in this study and ecotype I reference sequences, including human-derived sequences, is shown in Figure 2. The tree estimates that all 34 sequences identified in this study are located out with the cluster where the human sequences from the Unites States are present (shadowed). However, there is lack of a clear clustering for the human European and the sequences analysed in the present work. A second cluster is also defined, representing sequences from Croatia and Albania, derived from ovine, caprine, and mouflon (*Ovis gmelini*) samples.

## 4. Discussion

This study aimed to identify the *A. phagocytophilum* ecotypes present in 72 samples from UK ruminants by sequence-based typing of the *groEL* gene. Diversity in the *groEL* gene is one of the few markers used to differentiate between *A. phagocytophilum* ecotypes, and its use in this study allows comparison with other studies from different geographical regions. The sequence and phylogenetic analysis revealed the presence of a single *A. phagocytophilum* ecotype (ecotype I) in the ruminant samples confirming previous observations on the host and geographical restriction of these variants within Europe [22]. To the author’s knowledge, this is the first study describing *A. phagocytophilum* ecotypes in ruminant species in the UK. For data protection reasons, we are not able to disclose the precise location of individual samples across the UK. 

The initial primer selection, based on published information, allowed identification of the optimal primer combinations for the PCR assay. When two primer sets yielded identical results, we chose the combination offering the longest sequence to increase the probability of identifying *groEL* allelic polymorphism among isolates. The use of two primer sets allowed the preparation of a final consensus sequence for each sample based on four sequencing reactions, increasing accuracy, and eliminating potential PCR and sequencing errors. There was no difference in sequence quality in relation to host species or sample type, indicating that this method may be applied to samples from living or post-mortem cases. 

The *groEL* gene was amplified by one of the two PCR reactions in 69 of the 72 samples tested. Only three samples failed amplification in one of the two PCR reactions. The lack of amplification in these three samples indicates subtle differences in the performance of the two PCR reactions, which may be associated with unknown polymorphism in the primer binding sites and indicates that both approaches should be adopted if a negative result is obtained at the first attempt. Most nucleotide sequences obtained from the same sample were identical; however, three samples showed a single nucleotide difference between the two PCR reactions. This might indicate PCR errors and shows the need for confirmatory reactions when carrying out amplicon sequencing for ecotype identification. As two of the samples showed polymorphism at the same position, this is unlikely to be a PCR or sequencing artefact and could be due to a recent mutation within the host or the presence of two haplotypes associated with co-infection by two bacterial strains. Co-infection with multiple strains has previously been reported in roe deer and sheep [36]. 

Among the samples tested, we identified 13 single nucleotide polymorphisms or SNPs in different positions of the *groEL* gene, with some sequences presenting more than one. Our results show significant allelic diversity in the *groEL* gene, however, all nucleotide polymorphisms were synonymous, with no changes in the predicted amino acid sequence. *GroEL* is a highly conserved gene coding for an essential chaperon protein found in many bacteria and which, together with its co-chaperonin groES, plays an essential role in protein folding [37,38] and *Anaplasma*-tick interactions [39]. The protein is translocated into host cell nuclei, eventually altering the phenotype of the infected neutrophils, and the lack of non-synonymous substitutions suggests that the purifying selection is acting to maintain the essential function of this protein.

The prevalence and genotype of *A. phagocytophilum* strains in vector and host populations have been investigated in several countries to assess their zoonotic potential [7]. *Anaplasma phagocytophilum* infection has been identified throughout Europe in domestic ruminants, with variable rates according to the species. A seroprevalence of 80% was described in Norwegian sheep [40]. No *Anaplasma phagocytophilum* infection has been reported in the USA to date, suggesting that North American strains may not be very pathogenic for ruminants. In contrast, equine granulocytic anaplasmosis and granulocytic anaplasmosis in dogs are widely reported in the USA, South America, Europe, Asia, and Africa [7]. 

The *groEL* gene is recognised as an appropriate marker for phylogenetic analysis to distinguish between *A. phagocytophilum* ecotypes, host preference, and their pathogenicity, especially in relation to their zoonotic potential [15]. It is accepted that ecotype I represents strains present in several hosts, including zoonotic strains identified in humans and variants present in domestic animals [14,15,41], whereas ecotypes II, III, and IV do not include zoonotic strains and are mainly associated with ruminants, rodents, and non-human primates. Using *groEL* gene sequences, ecotype I was linked to red deer and ecotype II exclusively to roe deer in Norway [42], whereas in Poland, Germany, and the Czech Republic, the presence of all ecotypes except III [39,41,43] was detected in several host species, including roe deer, wild and domestic large ungulates, and brown hares [44,45]. All ecotypes were detected in Spain [2], Belgium, and the Netherlands in several domestic and wild species [14]. In Italy, a high prevalence of both ecotypes I and II was identified among wild ruminants and wild boars [16]. In France, using a different genetic marker belonging to the *ankA* gene cluster I, two different genetic groups were identified, mainly infecting humans and companion and farm animals [46]. Surveys have been conducted in countries in central and southern China, demonstrating that ecotype I is present in almost all Eurasia, whereas the remaining ecotypes (II–IV) have a more restricted distribution [47]. However, ecotype attribution often depends on the gene used to carry out the classification. For example, some strains detected in sheep, goats, cows, hedgehogs, wild carnivores, and other species might cluster into different genetic groups using different markers [7].

Despite the widespread presence of *A. phagocytophilum* in Europe, with varied rates of seropositivity recorded in different human populations (on average ~ 8.3%, reaching up to 31%) [48], human disease seems to be limited and less prevalent than in the United States, where it has been on the increase for some time [49]. This difference seems to be linked to presence of specific sub-clusters or haplotypes within ecotype I, showing different zoonotic potential ([7]). For example, Rar et al. [7], using *groEL* gene sequence analysis, described the presence of three different sub-clusters within ecotype I isolates. These included clusters containing sequences of (i) European isolates from horses, dogs, wild boars, wild and domestic ruminants, a raccoon, bears, and *I. ricinus*, (ii) American isolates from humans, horses, dogs, a cat, a rabbit, woodrats, and *I. pacificus* and (iii) a small Brazilian cluster with sequences derived only from dogs. Further clustering delineated a division between a European zoonotic group and North American group. Furthermore, according to Matei et al. [48] and based on the sequence of the 16S rRNA gene, North American strains seem to show less diversity compared to European strains and mainly belong to two variants, of which the Ap-ha represents the zoonotic group. Collectively, strains with clear zoonotic potential correspond to a minority of *A. phagocytophilum* sequences, and evidence is emerging indicating that a simple, one marker analysis is not sufficient to assign an isolate to a specific genogroup [7]. The attribution of zoonotic potential to a specific isolate is still work in progress and requires more epidemiological, clinical, and ecological information than phylogenetic analysis alone.

The results of the work presented here, albeit limited to a relatively small number of samples collected in three consecutive years, indicate the presence of ecotype I in UK ruminants and confirms that the ecotype distribution in the UK reflects closely the European situation. A recent UK tick survey [22] identified both ecotypes I and II, with a higher prevalence of ecotype I in ticks surveyed in different areas of England and Wales with the absence of ecotypes III and IV. The same study suggested a positive association between the presence of sheep in a specific area and a higher prevalence of *A. phagocytophilum*, rather than a specific environment. It is likely that the isolates typed in this work will represent strains of low zoonotic potential, similar to those already identified in the rest of Europe, as suggested by the phylogenetic analysis, where our sequences clustered independently of the North American zoonotic group. Lack of zoonotic potential seems also to be confirmed by the fact that very few instances of human granulocytic anaplasmosis have been reported in Europe [48]. However, it is unclear if this observation is due to lack of exposure in specific geographical areas, underreporting of the symptoms to medical practitioners, or to a true lack of pathogenicity even when exposure is high, as reported by a Swedish study [50]. Finally, the zoonotic importance of strains not associated with human disease and belonging to different ecotypes is unclear and requires further investigation to confirm their absence of pathogenicity. 

In summary, we have demonstrated the presence of an ecotype of *A. phagocytophilum* in the UK ruminant population closely clustering with strains identified in Europe with zoonotic potential. Its zoonotic potential should be considered until additional data are obtained. Meanwhile, better public information and medical awareness is required to limit exposure to tick bites as well as an epidemiological evaluation of the potential for *A. phagocytophilum* infection to cause immunosuppression in the human population. Definitive information on these strains’ correlation with pathogenicity and vector and host interaction, as well as serological and humoral immune responses, are still knowledge gaps that need to be filled. 

## Figures and Tables

**Table 1:** Relationship between *A. phagocytophilum* ecotypes, haplotypes, clusters, hosts, and vectors observed in distinct geographical locations in samples derived from different vectors and hosts. I. = ixodes; phylogenetic analysis based on the sequence of the *groEL* heat shock operon gene. From Jaarsma et al. [15], modified.

Ecotype	Cluster	Haplotypes (n)	Hosts	Vectors	Regions
Ecotype I	1	1095	Artiodactyla, Perisodactyla, Aves, Carnivora, Rodentia, Erinaceomorpha, Primates, Lagomorpha	*Ixodidae Lipoptena cervi*	Europe, Central America, and North America
	2	1	Artiodactyla	Unknown	Europe
Ecotype II	3	253	Artiodactyla, Carnivora, Rodentia	*Ixodidae Lipoptena cervi*	Asia, Europe, and South America
	4	31	Artiodactyla, Carnivora, non-human Primates, Rodentia	*Ixodidae*	Asia, Europe
Ecotype III	5	117	Rodentia, Soricomorpha	*Ixodidae*	Europe
	6	15	Rodentia,	*Ixodidae*	Europe
Ecotype IV	7	2	Aves, Erinaceomorpha, Rodentia	*Ixodidae*	Europe
	8	5	unknown	*I. ventalloi*	Europe

**Table 2:** Summary of the characteristics of the samples included in this study. APFG = *A. phagocytophilum* feral goat (positive control).

Species	Matrix	Total Number	Date Range
Ovine	Tissue	43	May 2020–Aug 2022
Ovine	EDTA blood	19	Jan 2022–Aug 2022
Bovine	Tissues	3	Jul 2020–Jun 2022
Bovine	EDTA blood	2	Jun 2022–Jul 2022
Cervine	EDTA blood	3	Feb 2022–Jun 2022
Caprine	Heparinised blood	1	Aug 2022
Originally isolated from caprine	Infected tick cell line APFG	1	May 2015

**Table 3:** Primers employed in the PCRs for the amplification and sequencing of the *groEL* gene, including primer specificities. In bold: degenerate bases, named according to the UIPAC coding system [30]. nd = non-degenerate.

Primer Name	Sequence	Specificity	Reference
groEL 569 F	ATGGTATGCAGTTTGATCGC	*A. platys*, *A. bovis, Ehrlichia* sp.	[26]
groEL nd643 F	ACTGATGGTATGCAGTTTGATCG	*A. phagocytophilum*	New
groEL 1236 R	TCTTT**R**CGTTC**Y**TT**M**AC**Y**TCAACTTC	*A. platys*, *A. bovis, Ehrlichia* sp., *Lentilitoribacter* sp., *Neorickettsia findlayensis*, *Rhodobiaceae bacterium*, *Erythrobacter* sp., *Cohaesibacter* sp.	[15]
groEl nd1236 R	TCTTTGCGTTCCTTCACCTCAACTTC	*A. phagocytophilum*	New

## Data Availability

Not applicable.

## Supplementary Materials

The following supporting information can be downloaded at: https://www.mdpi.com/article/10.3390/pathogens12020216/s1, Table S1: Nucleotide polymorphisms detected in the sequence of the groEL gene 571 bp fragment and predicted amino-acid changes. Table S2: Sequences used for phylogenetic analysis with duplicates removed (excel file). Figure S1: Alignment of the three consensus sequences showing polymorphism. Figure S2: Complete phylogenetic tree.
