# Supplementary material for: Presence of Anaplasma phagocytophilum Ecotype I in UK Ruminants and Associated Zoonotic Risk
Source: Pathogens. 2023 Jan 30;12(2):216. doi: 10.3390/pathogens12020216 (PMC9966478; doi:10.3390/pathogens12020216)
Supplement: Supplementary file 1 [file pathogens-12-00216-s001.zip › supplementary figure S1.pdf]

Alignment: C:\BioEdit\Temp\~out.tmp

```
               ....|....| ....|....| ....|....| ....|....| ....|....|
                        10         20         30         40         50
22_19 1pcr     CGGATATCTT TCGCCTTACT TTGTTACAAA TGCTGAAAAA ATGCTGGTGG
22_19 2pcr     CGGATATCTT TCGCCTTACT TTGTTACAAA TGCTGAAAAA ATGCTGGTGG
22_45 1pcr     CGGATATCTT TCGCCTTACT TTGTTACAAA TGCTGAAAAA ATGCTGGTGG
22_45 2pcr     TGGATATCTT TCGCCTTACT TTGTTACAAA TGCTGAAAAA ATGCTGGTGG
22_54 1pcr     ---------- ---------- ---------- ---------- -TGCTGGTGG
22_54 2pcr     CGGATATCTT TCGCCTTACT TTGTTACAAA TGCTGAAAAA ATGCTGGTGG


               ....|....| ....|....| ....|....| ....|....| ....|....|
                        60         70         80         90        100
22_19 1pcr     AATTTGAAAA TCCATACATA TTCCTTACTG AAAAGAAGAT TAATCTTGTA
22_19 2pcr     AATTTGAAAA TCCATACATA TTCCTTACTG AAAAGAAGAT TAATCTTGTA
22_45 1pcr     AATTTGAAAA TCCATACATA TTCCTTACTG AAAAGAAGAT TAATCTTGTA
22_45 2pcr     AATTTGAAAA TCCATACATA TTCCTTACTG AAAAGAAGAT TAATCTTGTA
22_54 1pcr     AATTTGAAAA TCCATACATA TTCCTTACTG AAAAGAAGAT TAATCTTGTA
22_54 2pcr     AATTTGAAAA TCCATACATA TTCCTTACTG AAAAGAAGAT TAATCTTGTA


               ....|....| ....|....| ....|....| ....|....| ....|....|
                       110        120        130        140        150
22_19 1pcr     CAAAGCATTC TACCAATCTT AGAAAACGTT GCACGGTCTG GAAGACCATT
22_19 2pcr     CAAAGCATTC TACCAATCTT AGAAAACGTT GCACGGTCTG GAAGACCATT
22_45 1pcr     CAAAGCATTC TACCAATCTT AGAAAACGTT GCACGGTCTG GAAGRCCATT
22_45 2pcr     CAAAGCATTC TACCAATCTT AGAAAACGTT GCACGGTCTG GAAGACCATT
22_54 1pcr     CAAAGCATTC TACCAATCTT AGAAAACGTT GCACGGTCTG GAAGACCATT
22_54 2pcr     CAAAGCATTC TACCAATCTT AGAAAACGTT GCACGGTCTG GAAGACCATT


               ....|....| ....|....| ....|....| ....|....| ....|....|
                       160        170        180        190        200
22_19 1pcr     GCTCATCATA GCTGAAGACG TTGAAGGTGA AGCTCTKAGC ACGCTTGTAC
22_19 2pcr     GCTCATCATA GCTGAAGACG TTGAAGGTGA AGCTCTTAGC ACGCTTGTAC
22_45 1pcr     GCTCATCATA GCTGAAGACG TTGAAGGTGA AGCTCTGAGC ACGCTTGTAC
22_45 2pcr     GCTCATCATA GCTGAAGACG TTGAAGGTGA AGCTCTGAGC ACGCTTGTAC
22_54 1pcr     GCTCATCATA GCTGAAGACG TTGAAGGTGA AGCTCTTAGC ACGCTTGTAC
22_54 2pcr     GCTCATCATA GCTGAAGACG TTGAAGGTGA AGCTCTGAGC ACACTTGTAC


               ....|....| ....|....| ....|....| ....|....| ....|....|
                       210        220        230        240        250
22_19 1pcr     TCAATAAGCT CCGTGGTGGC CTTCAAGTTG CTGCTGTAAA GGCGCCTGGT
22_19 2pcr     TCAATAAGCT CCGTGGTGGC CTTCAAGTTG CTGCTGTAAA GGCGCCTGGT
22_45 1pcr     TCAATAAGCT CCGTGGTGGC CTTCAAGTTG CTGCTGTAAA GGCGCCTGGT
22_45 2pcr     TCAATAAGCT CCGTGGTGGC CTTCAAGTTG CTGCTGTAAA GGCGCCTGGT
22_54 1pcr     TCAATAAGCT CCGTGGTGGC CTTCAAGTTG CTGCTGTAAA GGCGCCTGGT
22_54 2pcr     TCAATAAGCT CCGTGGTGGC CTTCAAGTTG CTGCTGTAAA GGCGCCTGGT
```

```
               ....|....| ....|....| ....|....| ....|....| ....|....|
                        260        270        280        290        300
22_19 1pcr     TTYGGTGACA GGAGAAAAGA CATGCTTGGC GATATTGCTG TAATAGTAGG
22_19 2pcr     TTCGGTGACA GGAGAAAAGA CATGCTTGGC GATATTGCTG TAATAGTAGG
22_45 1pcr     TTTGGTGACA GGAGAAAAGA CATGCTTGGC GATATTGCTG TAATAGTAGG
22_45 2pcr     TTTGGTGACA GGAGAAAAGA CATGCTTGGC GATATTGCTG TAATAGTAGG
22 54 1pcr     TTTGGTGACA GAAGAAAAGA CATGCTTGGC GATATTGCTG TAATAGTAGG
22_54 2pcr     TTTGGTGACA GGAGAAAAGA CATGCTTGGC GATATTGCTG TAATAGTAGG

               ....|....| ....|....| ....|....| ....|....| ....|....|
                        310        320        330        340        350
22_19 1pcr     CGCTAAGTAT GTAGTAAATG ACGAGCTTGC TGTTAAGATG GAAGATATCG
22_19 2pcr     CGCTAAGTAT GTAGTAAATG ACGAGCTTGC TGTTAAGATG GAAGATATCG
22_45 1pcr     CGCTAAGTAT GTAGTAAATG ACGAGCTTGC TGTTAAGATG GAAGACATCG
22_45 2pcr     CGCTAAGTAT GTAGTAAATG ACGAGCTTGC TGTTAAGATG GAAGACATCG
22 54 1pcr     CGCTAAGTAT GTAGTAAATG ACGAGCTTGC TGTTAAGATG GAAGATATCG
22_54 2pcr     CGCTAAGTAT GTAGTAAATG ACGAGCTTGC TGTTAAGATG GAAGATATCG

               ....|....| ....|....| ....|....| ....|....| ....|....|
                        360        370        380        390        400
22_19 1pcr     CTCTAAGCGA TCTTGGTACT GCTAAGAGCG TACGCATCAC AAAAGACGCA
22_19 2pcr     CTCTAAGCGA TCTTGGTACT GCTAAGAGCG TACGCATCAC AAAAGACGCA
22_45 1pcr     CTCTAAGCGA TCTTGGTACT GCTAAGAGCG TACGCATCAC AAAAGACGCA
22_45 2pcr     CTCTAAGCGA TCTTGGTACT GCTAAGAGCG TACGCATCAC AAAAGACGCA
22 54 1pcr     CTCTAAGCGA TCTTGGTACT GCTAAGAGCG TACGCATCAC AAAAGACGCA
22_54 2pcr     CTCTAAGCGA TCTTGGTACT GCTAAGAGCG TACGCATCAC AAAAGACGCA

               ....|....| ....|....| ....|....| ....|....| ....|....|
                        410        420        430        440        450
22_19 1pcr     ACTACTATCA TAGGTAGTGT TGATAGCAGT TCTGAAAGCA TAGCTAGCAG
22_19 2pcr     ACTACTATCA TAGGTAGTGT TGATAGCAGT TCTGAAAGCA TAGCTAGCAG
22_45 1pcr     ACTACTATCA TAGGTAGTGT TGATAGCAGT TCTGAAAGCA TAGCTAGCAG
22_45 2pcr     ACTACTATCA TAGGCAGTGT TGATAGCAGT TCTGAAAGCA TAGCTAGCAG
22 54 1pcr     ACTACTATCA TAGGTAGTGT TGATAGCAGT TCTGAAAGCA TAGCTAGCAG
22_54 2pcr     ACTACTATCA TAGGTAGTGT TGATAGCAGT TCTGAAAGCA TAGCTAGCAG

               ....|....| ....|....| ....|....| ....|....| ....|....|
                        460        470        480        490        500
22_19 1pcr     GACTAATCAA ATCAAAGCTC AGATAGAAAA TTCTAGTTCT GATTATGACA
22_19 2pcr     GACTAATCAA ATCAAAGCTC AGATAGAAAA TTCTAGTTCT GATTATGACA
22_45 1pcr     GACTAATCAA ATCAAAGCTC AGATAGAAAA TTCTAGTTCT GATTATGACA
22_45 2pcr     GACTAATCAA ATCAAAGCTC AGATAGAAAA TTCTAGTTCT GATTATGACA
22 54 1pcr     GACTAATCAA ATCAAAGCTC AGATAGAAAA TTCTAGTTCT GATTATGACA
22_54 2pcr     GACTAATCAA ATCAAAGCTC AGATAGAAAA TTCTAGTTCT GATTATGACA
```

```
               ....|....| ....|....| ....|....| ....|....| ....|....|
                        510        520        530        540        550
22_19 1pcr     AGGAAAAGCT TAGAGAACGT TTAGCAAAGC TTTCCGGTGG TGTTGCTGTA
22_19 2pcr     AGGAAAAGCT TAGAGAACGT TTAGCAAAGC TTTCCGGTGG TGTTGCTGTA
22_45 1pcr     AGGAAAAGCT TAGAGAACGT TTAGCGAAGC TTTCCGGTGG TGTTGCTGTA
22_45 2pcr     AGGAAAAGCT TAGAGAACGT TTAGCAAAGC TTTCCGGTGG TGTTGCTGTA
22 54 1pcr     AGGAAAAGCT TAGAGAACGT TTAGCGAAGC TTTCCGGTGG CGTTGCTGTA
22_54 2pcr     AGGAAAAGCT TAGAGAACGT TTAGCAAAGC TTTCCGGTGG CGTTGCTGTA
```

```
               ....|....| ....|....| .
                        560        570
22_19 1pcr     CTCAAGGTTG GTGGATCCAG C
22_19 2pcr     CTCAAGGTTG GTGGATCCAG C
22_45 1pcr     CTCAAGGTTG GTGGATCCAG C
22_45 2pcr     CTCAAGGTTG GTGGATCCAG C
22 54 1pcr     CTCAAGGTTG GTGGATCCAG C
22_54 2pcr     CTCAAGGTTG GTGGATCCAG C
```
