# Supplementary material for: Presence of Anaplasma phagocytophilum Ecotype I in UK Ruminants and Associated Zoonotic Risk
Source: Pathogens. 2023 Jan 30;12(2):216. doi: 10.3390/pathogens12020216 (PMC9966478; doi:10.3390/pathogens12020216)
Supplement: Supplementary file 1 [file pathogens-12-00216-s001.zip › supplementary table S1.pdf]

Supplementary Table S1. Nucleotide polymorphisms detected in the sequence of the *groEL* gene 571 bp fragment and predicted amino-acid changes in the samples analysed in this study. Nucleotide position refers to the position in the amplified fragment once primers were removed; reference base represent the base identified as the most common in the overall alignment and called base represent the nucleotide substitution observed in the same position; number of sequences indicates the number of the sequences with the reference base; SNP indicates number of sequences showing base change at that specific position; Underlined are the base changes in each codon.

Nucleotide position	Ref base	Called base	Possible codons modified		Coded amino-acid	Type of mutation	Number of sequences showing SNPs
1 (588)	C	Y	CGC	CGT	Arg	Synonymous	1/72
10 (597)	T	A	CTT	CTA	Leu	Synonymous	1/72
73 (660)	C	T	TTC	TTT	Phe	Synonymous	1/72
145 (732)	A	G	AGA	AGG	Arg	Synonymous	36/72
187 (774)	G	T	CTG	CTT	Leu	Synonymous	30/72
193 (780)	G	A	ACA	ACG	Thr	Synonymous	6/72
253 (840)	T	C	TTT	TTC	Phe	Synonymous	20/72
262 (849)	G	R	AGG	AGA	Arg	Synonymous	1/72
346 (933)	T	C	GGT	GGC	Gly	Synonymous	33/72
415 (1002)	T	C	GGT	GGC	Gly	Synonymous	22/72
526 (1113)	G	A	GCG	GCA	Ade	Synonymous	23/72
535 (1122)	C	T	TCC	TCT	Ser	Synonymous	10/72
541 (1128)	C	T	GGC	GGT	Gly	Synonymous	22/72
