# Supplementary figures and images for: Presence of Anaplasma phagocytophilum Ecotype I in UK Ruminants and Associated Zoonotic Risk
Source: Pathogens. 2023 Jan 30;12(2):216. doi: 10.3390/pathogens12020216 (PMC9966478; doi:10.3390/pathogens12020216)

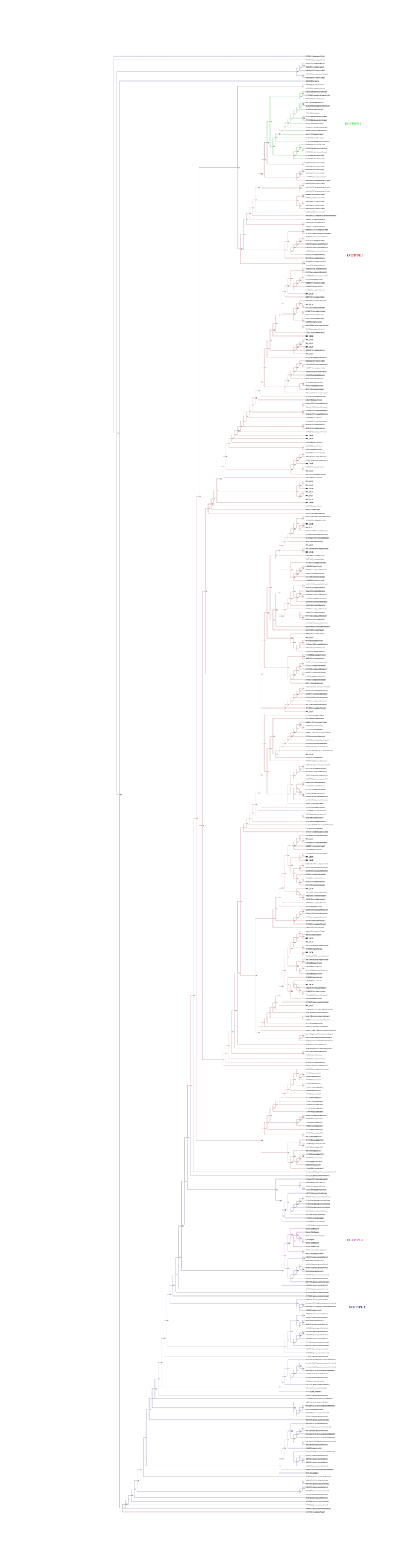

Supplement: Supplementary file 1 [file pathogens-12-00216-s001.zip › supplementary figure S2 large tree.jpg]
